# Microscopic and Molecular Identification of *Cyclospora cayetanensis* and *Cystoisospora belli* in HIV-Infected People in Tabriz, Northwest of Iran

**DOI:** 10.3390/tropicalmed8070368

**Published:** 2023-07-17

**Authors:** Saba Ramezanzadeh, Gholamreza Barzegar, Hamid Owaysee Osquee, Majid Pirestani, Mahmoud Mahami-Oskouei, Maryam Hajizadeh, Seyed Abdollah Hosseini, Sonia M. Rodrigues Oliveira, Mahmoud Agholi, Maria de Lourdes Pereira, Ehsan Ahmadpour

**Affiliations:** 1 Infectious and Tropical Disease Research Center, Tabriz University of Medical Sciences, Tabriz 51666-14766, Iran; saba.ra94@yahoo.com (S.R.); rezabarzegar51@gmail.com (G.B.);; 2 Department of Parasitology and Mycology, Tabriz University of Medical Sciences, Tabriz 51666-14766, Iran; 3 Department of Parasitology, Faculty of Medical Sciences, Tarbiat Modares University, Tehran 331-14115, Iran; 4 Drug Applied Research Center, Tabriz University of Medical Sciences, Tabriz 51666-15731, Iran; 5 Immunology Research Center, Tabriz University of Medical Sciences, Tabriz 51666-15731, Iran; 6 Department of Parasitology and Mycology, Faculty of Medicine, Mazandaran University of Medical Sciences, Sari 48157-33971, Iran; 7 CICECO—Aveiro Institute of Materials, University of Aveiro, 3810-193 Aveiro, Portugal; 8 Hunter Medical Research Institute, New Lambton, NSW 2305, Australia; 9 Department of Parasitology and Mycology, School of Medicine, Fasa University of Medical Sciences, Fasa 74616-86688, Iran; 10 Department of Medical Sciences, University of Aveiro, 3810-193 Aveiro, Portugal

**Keywords:** intestinal parasites, prevalence, Tabriz, *Cyclospora cayetanensis*, *Cystoisospora belli*

## Abstract

Opportunistic pathogens such as *Cryptosporidium*, *Cystoisospora belli*, and *Cyclospora cayetanensis* cause various gastrointestinal and non-digestive disorders in people with HIV/AIDS. These symptoms are especially severe in HIV-infected people who have a CD4+ count of less than 200 cells/mL. This study aimed to determine the prevalence of *C. belli* and *C. cayetanensis* infections among people living with HIV in Tabriz, northwest of Iran. This descriptive study was performed on 137 people with HIV who had been referred to behavioral disease counseling centers in Tabriz. Then, after receiving written consent, fecal samples were collected and evaluated for the detection of parasitic infections using direct methods and modified acid fast staining, as well as polymerase chain reaction (PCR).From the 137 fecal samples collected (98 males and 39 females, between 20 and 40 years old), 1.5% were positive for *C. cayetanensis* and 2.9% were positive for *C. belli*. Due to the prevalence of *C. cayetanensis* and *C. belli* in people with HIV in Tabriz, essential measures, including personal hygiene training for infection control and prevention, seem necessary.

## 1. Introduction

Intestinal parasitic infections (IPIs) are a serious public health problem in developing countries, such that some parasitic infections are neglected tropical diseases [1,2]. Numerous parasites, including protozoa (e.g., *Entamoeba histolytica*, *Giardia intestinalis*, and *Cryptosporidium* spp.) and helminths (e.g., *Ascaris lumbricoides*, *Trichuris trichiura*, *Hymenolepis nana*, *Necator americanus*, and *Ancylostoma duodenale*), can cause gastrointestinal infections. The burden of IPIs is estimated at three billion people worldwide; however, their prevalence is likely higher than the number of reported cases because their symptoms are not clear in the early stages of infection [3,4]. The IPIs are closely related to poverty, unsafe drinking water, poor sanitation and hygiene, with symptoms including diarrhea, anemia, malabsorption, weight loss, abdominal pain, dyspepsia, growth retardation, and delayed intellectual development [2,5].

After the advent of human immunodeficiency virus (HIV) and acquired immunodeficiency syndrome (AIDS) in the world, intestinal parasites causing benign and common infections between humans and animals became opportunistic and began to cause gastrointestinal and systemic symptoms, including severe diarrhea, in people living with HIV and/or AIDS (PLWHA) [6,7,8]. Opportunistic parasitic pathogens include *Cyclospora cayetanensis*, *Cryptosporidium parvum, Cystoisospora belli* (Apicomplexa protozoans), *Strongyloidesstercolaralis*, *Giardia intestinalis*, *Entamoeba histolytica*, and *Blastocystishominis* [9,10,11,12]. These pathogens can cause signs and symptoms such as watery diarrhea, loss of appetite, weight loss, abdominal cramps, nausea, fever, headache, and vomiting [7,13]. However, the severity of the infection depends on parasite factors (parasite species, parasite load, duration of infection, and co-infections), host factors (habitat, gender, age, nutritional and immunological status), and socioeconomic factors [14]. Research shows that 30% to 60% of people living with HIV/AIDS (PLWHA) in developed countries and 90% of PLWHA in low and middle-income countries have diarrhea, and IPIs are a major cause of this symptom in these populations [15,16]. IPIs usually aggravate the clinical conditions of immunocompromised patients and cause resistant infections. These infections can play an important role in the progression of HIV infection [17,18]. 

So far, more than 20 *Cyclospora* species have been identified in humans and various animals, including snakes, rodents, and monkeys, of which *Cyclospora cayetanensis* (*C. cayetanensis*) is the only species that infects humans [19,20]. *C. cayetanensis* infection has recently emerged as one of the most opportunistic infections with global distribution, and is also associated with foodborne outbreaks [21]. This coccidian parasite is an obligate intracellular apicomplexan that infects the epithelial cells of the small intestine and causes gastrointestinal disease. Most of the transmission of this infection occurs through the consumption of contaminated food, particularly fresh and raw produce such as fruits and vegetables, or water contaminated with oocysts [22,23]. *C. cayetanensis* oocysts may survive in the environment for long periods of time. They are also resistant to many disinfectants, such as chlorination. Although the route of infection is fecal–oral, the transmission from person to person has not been clearly established, as oocysts require 7 to15 days to sporulate and mature into their infectious forms [24]. This organism infects the upper intestine, causing flattening of the villi, crypt hyperplasia, inflammation, and malabsorption [25]. Based on the results of previous studies, the prevalence of *C. cayetanensis* is generally 0–13% in immunocompetent people with diarrhea and 0 to 36% in immunodeficient people [19]. *Cystoisospora belli* (formerly known as *Isospora belli*), such as *C. cayetanensis,* is a coccidian, unicellular protozoan parasite. *C. belli* occurs globally, but is more common in tropic and subtropical regions. The life cycle of *C. belli* is similar to that of *C. cayetanensis*. This parasite enters the body of the host along with contaminated water or food and causes disease [26,27]. It is also suggested that the sporulated oocysts could be mechanically transmitted by arthropods [28]. *C. belli* is another opportunistic pathogen that causes asymptomatic or self-limiting diarrhea in healthy people, but in PLWHA, it causes severe chronic diarrhea and malabsorption that may lead to wasting syndrome or even death [29]. In some patients, relapse and chronic infection may occur despite the presence of an efficient immune system [27].

*C. cayetanensis* and *C. belli* infections have emerged as significant public health concerns worldwide. Additionally, in developed countries, these diseases have been associated with cases of traveler’s diarrhea. Furthermore, *C. cayetanensis* and *C. belli* may be present along with other co-infecting parasites. As mentioned, in immunocompetent hosts, cyclosporiasis and cystoisosporiasis can be asymptomatic, or can cause mild to moderate self-limiting diarrhea. On the other hand, in immunocompromised and immunosuppressed hosts, severe intestinal damage and protracted or chronic watery diarrhea, along with nausea, abdominal pain, and fever, may be observed. Therefore, infection prevention is important for at-risk groups such as AIDS patients [21,27]. Unfortunately, in the absence of anti-retroviral therapy (ART), PLWHA suffer severely from the effects of opportunistic parasitic infections. These consequences are especially greater in people who have very low CD4^+^T cell counts, and can even cause death [30]. Despite reports of infection from different regions of Iran [31], there is not enough information about the prevalence of opportunistic parasites such as *C. cayetanensis* and *C. belli* among PLWHA in northwest of Iran. Hence, due to the impact of these parasites on reducing the quality of life of PLWHA, we aimed to determine the prevalence of *C. cayetanensis* and *C. belli* among PLWHA in Tabriz. Therefore, appropriate strategies will be provided for the timely treatment and effective prevention of parasitic opportunistic pathogens.

## 2. Materials and Methods

### 2.1. Study Area

Tabriz is a city in Iran, located west of the East Azerbaijan province. It has an area of 24,451 square kilometers, and is the 5th most populous city in Iran and the 338th most populous city in the world [32]. Whilst the prevalence of HIV among the general population of Iran remains low, the numbers are significant, particularly among users of injected drugs (4.32% of injectable drug addicts in 2019). Iran counted 59,314 HIV-positive people in 2019 (of these, 862 were children under 15 years old; 15,501 were women over 15 years; and 42,952 were men over 15 years), facing an increase in infections and HIV-related deaths [UNAIDS, 2020; see: https://www.unaids.org/sites/default/files/country/documents/IRN_2020_countryreport.pdf (accessed on 14 November 2020)].

### 2.2. Study Design and Population

This descriptive study was conducted on a population of HIV-infected patients who were registered for care and treatment at behavioral disease counseling centers in *Tabriz*. This study lasted from October 2019 to December 2021. Simple random sampling methods were used to collect samples. All individuals received anti-retroviral therapy (ART). This study was approved by the Ethics Committee of Tabriz University of Medical Sciences (REC.1400.320). Informed written consent was provided by the patients prior to sample collection. The patients’ privacy and confidentiality were respected; a code was used instead of the names of the individuals and the patients’ genders, age ranges, occupations, levels of education, places of residence, and economic statuses were recorded. Only PLWHA were included in the study, and there was no age or sex restriction to enter the study. 

### 2.3. Laboratory Procedures

One stool sample was taken from each patient and evaluated for the presence of blood, as well as for the stool form. After collection, the samples were divided into two parts in sterile labeled containers and immediately transferred to the parasitology laboratory of Tabriz Medical University for the detection of intestinal coccidian oocysts (*C. cayetanensis* and *C. belli*). The second parts of the samples were stored at −20 until molecular analysis.

### 2.4. Parasitology Detection Procedure

For direct smear examinations, all collected fecal samples were examined by saline and iodine wet mounts within 6 h after collection. At first, one drop of normal saline (0.85% NaCl) was added onto a microscopy slide by mixing it with a small amount of the sample (approximately 2 mg). The prepared smear was covered with a cover slide and examined under 10× and 40× objectives. Afterward, the stool samples were mixed by adding a drop of Lugol’s iodine solution and re-examined [33,34]. 

Another part of the same sample was examined using the formol ether concentration technique. The procedure was as follows: approximately 1–2 g of the stool sample of each subject was suspended with normal saline solution, filtered through gauze, and centrifuged at 800× *g* for 5 min. The sediment was suspended with 10 mL of 10% formalin and mixed thoroughly. After 20 min, 3 mL of ether was added, and the mixture was properly shaken for 30 s. The tube was again centrifuged at 800× *g* for 5 min, the supernatant was discarded, and the sediment was used for microscopic examination by two well-trained microscopists for the presence of helminths and intestinal protozoa. Sterile screw cap vials were used to prevent the contamination of the samples with water, soil, or urine.

### 2.5. Modified Acid Fast Technique

Smears were prepared from the fresh and concentrated samples on a clean glass slide. Air-dried smears were fixed by heating. Alcohol fixation was used for the fresh samples. Then, the smears were covered with carbol fuchsin stain and heated gently (boiling was avoided) for approximately 5 min. If required, more stain was added and the stain was prevented from drying. The slides were rinsed with clean water, which continued until the runoff became clear and colorless. For decolorization, the smears were covered with 3% *v/v* acid alcohol for approximately 30 s until they were sufficiently decolorized, and subsequently washed with clean water. Afterward, the slides were covered with methylene blue stain for 1 min and the stains were washed and air-dried. Finally, the slides were observed microscopically (Olympus BX41TF light microscope with a coupled camera) using the 100× oil immersion objective [9,31].

### 2.6. DNA Extraction and PCR

To extract DNA from fecal samples, a kit from Favorgen Company (Cat No. FASTI 001, Ping Tung, Taiwan) was used, and tests performed according to the instructions. The extracted DNA samples were kept frozen at −20 °C for further use. A polymerase chain reaction (PCR) was performed using primers specific to the region coding for small subunit ribosomal RNA genes (SSU rRNA) of *C. cayetanensis* with 116 bp amplicon (forward primer: 5′-GCAGTCACAGGAGGCATATATCC-3′; reverse primer: 5′-ATGAGAGACCTCACAGCCAAAC-3′) [35]. The amplification program began with an initial denaturation step at 95 °C for 5 min, followed by a 40-cycle program of 95 °C for 30 s, 59 °C for 30 s, and 72 °C for 30 s, and at the end, a final extension step was carried out at 72 °C for 5 min.

For the molecular diagnosis of *C. belli*, we used a 175 bp fragment of an 18S ribosomal RNA gene with forward primer 5′-CCGAACGTCATCCGAAATAG-3′ and reverse primer 5′-ACTAGGAGCTGACGATACAC-3′ [31]. The amplification program was similar to SSU rRNA except for the annealing temperature (58 °C for 30 s). All PCR products were separated by agarose (1%) gel electrophoresis containing ethidium bromide (0.2 µg/mL), and then visualized on a UV transilluminator. 

### 2.7. Genotyping and Phylogenetic Analysis

The positive samples were sequenced in both directions (forward and reverse) by Gen Fanavaran (Tehran, Iran). Next, the sequence of the obtained samples was edited using SequencherTmv.4.1.4 and Chromas version 2.33 [http://www.technelysium.com.au/chromas.html (accessed on 18 May 2022)] software. The phylogenetic analysis was performed by constructing gene trees using the neighbor-joining (NJ) method in molecular evolutionary genetics analysis (MEGA) software, version 5.0 [36]. 

### 2.8. Statistical Analysis

The results were tabulated using Excel 2019 software, and IBM SPSS 26.0 software was used for data analysis (to determine the presence or absence of a relationship between parasite prevalence and demographical factors). The default level, with *p* < 0.05, was considered statistically significant.

## 3. Results

**Figure 1:**
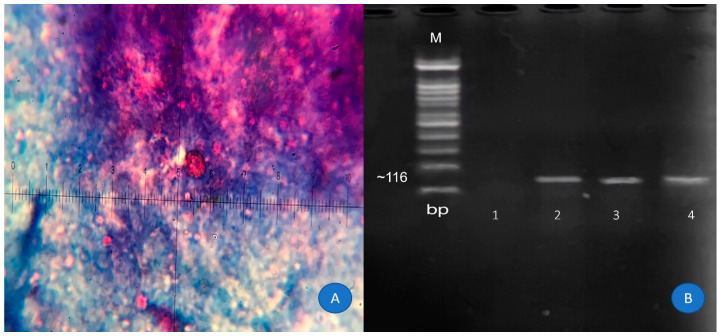
Identification of *C. cayetanensis* oocysts. (**A**) Non-sporulated oocysts of *C. cayetanensis* in modified acid fast staining (100×). (**B**) Agarose gel of PCR products. Lane M, 100 bp molecular size marker; lane 1: negative control, lanes 2,3: positive cases, lane 4: positive control.

**Figure 2:**
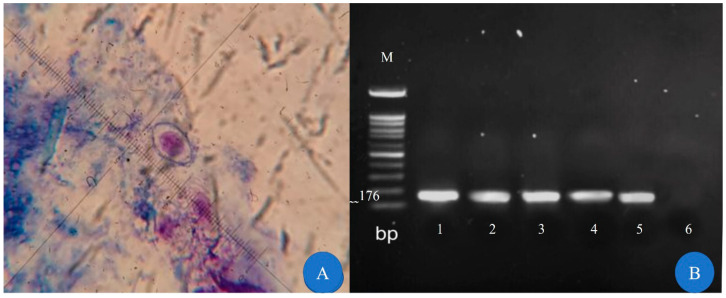
Identification of *C. belli* oocysts. (**A**) Non-sporulated oocysts of *C. belli* in modified acid fast staining (100×). (**B**) Agarose gel of PCR products. Lane M, 100 bp molecular size marker; lane 1: positive control, lanes 2–5: positive cases, and lane 6: negative control.

A total of 137 patients participated in the present study. All individuals were infected with HIV, and the sample included 39 (28.5%) women and 98 (71.5%) men who agreed with the study’s research. Most of the patients were in the age range of 20–40 years. All the participants who completed the questionnaire had the necessary cooperation. The questionnaire included questions about gender, age range, place of residence, income, education, and occupation of the participant. The demographic information of the participants is described in Table 1. All samples were examined by direct methods, i.e., modified acid fast staining and PCR. Fortunately, the prevalence rate of these intestinal parasites was not high in the studied population: five suspected cases of *C. cayetanensis* and one suspected case of *C. belli* were identified by means of the staining method. Based on the molecular method, two (1.5%), and four (2.9%) cases were confirmed to beinfected with *C. cayetanensis* and *C. belli*, respectively. Also, 33 (24.1%) cases of *Blastocystis hominis*, 8 (5.8%) cases of *Giardia lamblia*, and 20 (14.6%) cases of *Cryptosporidium* spp. were detected in the stool samples. *C. cayetanensis* was identified with non-sporulated oocysts measuring 8–10 µ. The results of the staining and PCR methods for *C. cayetanensis* detection are shown in Figure 1. *C. belli* was also identified as non-sporulated and oval oocysts with sizes of 20–25 µ. The results of the staining and PCR methods of *C. belli* detection are shown in Figure 2.

**Figure 3:**
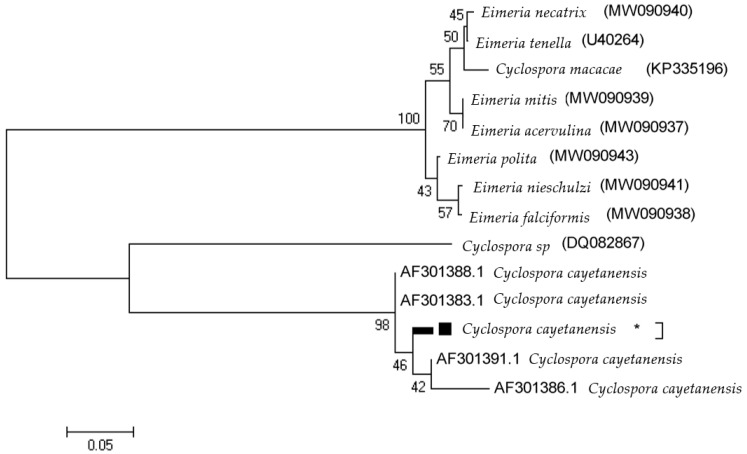
The phylogenetic tree based on *C. cayetanensis* small subunit ribosomal RNA nucleotide sequences from individuals with *C. cayetanensis* infection, as well as those corresponding to different *C. cayetanensis* genotypes taken from the GenBank database. Bootstrap values ≥ 70, achieved after 1000 replicates, are shown at the nodes. Isolated sequences in this study are indicated by “*”.

**Figure 4:**
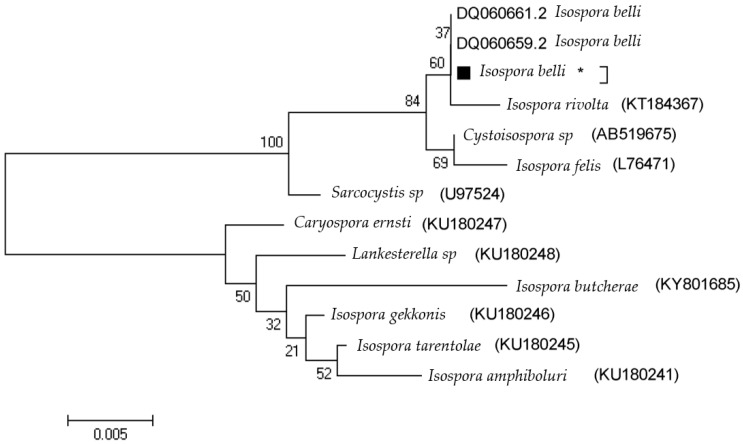
The phylogenetic tree based on *C. belli* 18S ribosomal RNA nucleotide sequences from individuals with *C. belli* infection, as well as those corresponding to different *C. belli* genotypes taken from the GenBank database. Bootstrap values ≥ 70, achieved after 1000 replicates, are shown at the nodes. Isolated sequences in this study are indicated by “*”.

The cases of *C. cayetanensis* included one woman and one man, one of whom had a lower CD4 cell count of 500 cells/mL and the other who had a count of more than 500 cells/mL. In cases of *C. belli* infection, which included two men and two women, only one of the cases had a CD4 count of fewer than 500 cells/mL. The relationships between *C. cayetanensis* and *C. belli* infections and the factors in the questionnaire are shown in Table 1. The positive PCR products were analyzed for molecular analysis just for the purpose of determining sequences, recording genes, and drawing phylogenetic charts (Figure 3 and Figure 4). The *C. cayetanensis* isolated from patients in Nepal (AF301383 and AF301386) was96% identical to the *C. cayetanensis* detected in the current study. Furthermore, the *C. belli* isolated from a patient in Thailand (DQ060661) was 95% identical to the *C. belli* detected in the current study.

## 4. Discussion

HIV/AIDS patients are highly susceptible to opportunistic pathogens, which has become a major public health problem with adverse consequences for patients and health systems. There is insufficient information available on the prevalence of parasitic infections, particularly *C. Cayetanensis* and *C. belli* parasites, among PLWHA in Iran. In the present study, we demonstratedthat the prevalence rates of *C. cayetanensis* and *C. belli* parasites in PLWHA were 1.5% and 2.9%, respectively, in Tabriz, northwest of Iran. PLWHA are more exposed to infections and their complications due to their immune system defects [22]. 

Diarrhea is one of the most common and important diseases in immunocompromised patients, as well as in children. Due to insufficient immune responses, the clinical symptoms caused by these infections in the aforementioned risk groups become more severe and can even be life-threatening. In underdeveloped countries, the incidence and mortality of acute diarrhea are higher in children. Additionally, in developing and underdeveloped countries, due to the lack of safe sources of drinking water, poor sanitation, poverty, and underdeveloped public health services, the complications are multiplied. Considering the high prevalence of HIV in these countries, AIDS-associated diarrhea can have irreparable consequences for these patients. 

Diarrhea is defined as the passage of three or more loose or liquid stools per day (or more frequent passage than is normal for the individual). It is usually a symptom of gastrointestinal infection caused by a variety of microorganisms, such as parasites, which may spread through contaminated food or drinking water or from person to person as a result of poor hygiene [37,38]. According to the World Health Organization, classic diarrhea is generally differentiated into acute and chronic based on its duration. Acute diarrhea is described as having acute onset and duration of no more than 14 days, whereas chronic or persistent diarrhea is defined as having a duration of more than 14 days [39]. In addition to rehydration, the key point in the treatment of diarrhea is the treatment of the infectious agent. Effective treatment would be useful as an adjunct to ART, especially in situations where antiretroviral drugs are either too expensive or unavailable [40,41,42]. However, AIDS-associated deaths have decreased dramatically with highly active antiretroviral therapy in areas of the world that can afford these treatments. Surprisingly, in this study, none of the participants had diarrhea, and had a soft or formed consistency. Several factors could have caused the absence of symptoms of diarrhea in these patients. Certainly, the most important reason may be the continuous screening of these patients by the health care system. On the other hand, the use of ART drugs can also reduce gastrointestinal symptoms, especially diarrhea. Although there were no patients with diarrhea in this study, the presence of chronic parasitic infections indicates the need for greater caution in these patients. 

Parasitic diseases are also a health problem for patients. Intestinal parasitic infections are common infectious diseases worldwide, considered to be neglected tropical diseases which lead to significant morbidity and mortality. Intestinal parasites can disrupt the absorption and function of the intestinal barrier by inflicting acute epithelial damage, leading to mild to severe diarrhea. Since diarrhea and diseases caused by parasitic infections with other opportunistic pathogenic infections, such as bacteria, are often misdiagnosed, the correct diagnosis and treatment of these people should be apriority of health and treatment centers. In the present study, the number of male participants was about twice that of female participants, and most of the patients were in the age range of 20–40 years old. Moreover, most of the individuals were urban dwellers with low or medium income levels. Most of these people had received less than a diploma in terms of education and were self-employed or unemployed. The relationship between these factors and infection with the corresponding parasites in PLWHA was investigated, and no significant difference between the factors and the prevalence of the infections was observed. It is believed that this result is due to the limited sample size, limited number of positive samples observed, and the similarities in the living standards of PLWHA.

In a systematic study conducted in 2022, the global prevalence of the *C. cayetanensis* parasite in PLWHA was 3.5%. The highest prevalence of this protozoan was reported in South America [23]. In another systematic review that was conducted in 2018, the overall prevalence of *C. belli* in PLWHA was found to be 2.5%. According to this review, the highest prevalence of *C.* belli was observed in low-income countries [26]. It seems that the prevalence of these parasites is more than the reported cases due to the lack of diagnostic tools. However, in immunocompromised and immunosuppressed patients, such infections are opportunistic and should be given more attention.

A study was conducted to investigate the prevalence of parasitic infections of *C. cayetanensis* and *C. belli* in 87 patients with colorectal cancer in Lorestan Province, Iran. A modified acid fast staining method was used for diagnosis. The prevalence of infection with *C. cayetanensis* was 5.74%, infection with *C. belli* was 9.2%, and co-infection was 3.44% [43].In another study in Tehran, 102 PLWHA were examined for parasitic infections. Molecular and staining methods were used for diagnosis, and 1 case of *C. cayetanensis* was identified [22].In general, little and incomplete information is available on the prevalence of *C. cayetanensis* and *C. belli* parasitic infections in Iran.

Direct examination, staining, and molecular methods can be used to detect intestinal parasites. However, the wet mount microscopic method is widely used for diagnosis in clinical stool samples. Smears can be prepared directly from fresh stool or after the concentration procedure, which increases the possibility of detecting small numbers of parasites in samples. Furthermore, some parasites are shed discontinuously, so consecutive stool samples should be collected and examined for accurate parasite detection. Thus, the correct diagnosis by means of the direct and staining methods depends on ability and sufficient experience, so there is a possibility of misdiagnosis. However, molecular methods such as PCR have high sensitivity and accuracy. We used all three methods for diagnosis. It seems that, in some studies, only direct and staining methods have been used to detect these parasites, and a higher percentage of parasitic infections have been reported due to human error. For example, in a study conducted in Ghana in 2018, the prevalence of *C. cayetanensis* in HIV-infected people was reported to be 32% [44]. In this study, the modified acid fast staining method was used for diagnosis. Therefore, it seems that the use of molecular methods with high accuracy and sensitivity is more suitable [45]. 

On the other hand, with the development of antiviral drugs in developed countries, the prevalence of opportunistic infections has decreased significantly. In the current study, all participants were receiving ART, which in turn reduced the parasitic infection. Among the limitations of the study, it can be mentioned that all patients received ART, and it was not possible to compare the prevalence of parasites in people who received the drug regimen with people who did not receive the drug. Also, accurate CD4 cell counts were not available for all patients. To determine the prevalence of parasitic infections and to control and reduce these infections, it is recommended that the prevalence of parasitic infections in different geographical regions of the country be checked by direct and molecular methods in people with impaired immune systems in a study with a larger sample size.

## 5. Conclusions

The results of the present study indicate the presence of *C. cayetanensis* and *C. belli* parasites in the region and suggest that these infections may be more common than is currently acknowledged in developing countries. Also, additional studies are needed to identify any potential endemic foci in different regions. Personal health education and preventive measures to avoid parasitic infections are necessary for these proud people, and it is recommended that HIV patients with a CD4 cell count of less than 200 cells/mL should be considered for early and periodic screening. In this sense, our research group has ongoing training action plans aimed at the community in general.

## Figures and Tables

**Table 1:** Correlation of the desired criteria in the questionnaire with *C. cayetanensis* and *C. belli* (*p* < 0.05).

Variables		*Cyclospora cayetanensis*		*p* Value	*Cystoisospora belli*		*p* Value
		Positive	Negative		Positive	Negative	
Gender	Female	1	38	0.2501	2	37	0.1682
	Male	1	97		2	96	
Age	<20 20–40 40–60 >60	0 1 1 0	2 79 53 1	0.3893	0 3 1 0	2 77 53 1	0.2926
Residence	Urban	2	128	0.3716	4	126	0.3203
	Rural	0	7		0	7	
Economic status	Poor (<USD 400/month) Average (USD400–2000/month) Rich (>USD 2000/mounth)	0 2 0	64 66 5	0.1401	2 2 0	62 66 5	0.4023
Education	Illiterate Under high school degree High school degree University degree	0 1 0 1	66 78 14 17	0.0939	1 2 0 1	25 77 14 17	0.4326
Job	Student Freelance Employee Housekeeper Unemployed	0 2 0 0 0	8 54 2 28 43	0.0958	0 1 0 2 1	8 55 2 26 42	0.2644

## Data Availability

The data supporting the findings of this study can be obtained from the corresponding author on reasonable request.

## References

[1] Wegayehu T., Tsalla T., Seifu B., Teklu T. (2013). Prevalence of intestinal parasitic infections among highland and lowland dwellers in Gamo area, South Ethiopia. BMC Public Health.

[2] Daryani A., Hosseini-Teshnizi S., Hosseini S.-A., Ahmadpour E., Sarvi S., Amouei A., Mizani A., Gholami S., Sharif M. (2017). Intestinal parasitic infections in Iranian preschool and school children: A systematic review and meta-analysis. Acta Trop..

[3] World Health Organization (2013). Sustaining the Drive to Overcome the Global Impact of Neglected Tropical Diseases: Second WHO Report on Neglected Diseases.

[4] Norhayati M., Fatmah M., Yusof S., Edariah A. (2003). Intestinal parasitic infections in man: A review. Med. J. Malays..

[5] Wong L.W., Ong K.S., Khoo J.R., Goh C.B.S., Hor J.W., Lee S.M. (2020). Human intestinal parasitic infection: A narrative review on global prevalence and epidemiological insights on preventive, therapeutic and diagnostic strategies for future perspectives. Expert Rev. Gastroenterol. Hepatol..

[6] White N. (2014). Malaria. Manson’s Tropical Infectious Diseases.

[7] Barcelos N.B., Silva L.d.F., Dias R.F.G., Menezes Filho H.R.d., Rodrigues R.M. (2018). Opportunistic and non-opportunistic intestinal parasites in HIV/AIDS patients in relation to their clinical and epidemiological status in a specialized medical service in Goiás, Brazil. Rev. Do Inst. De Med. Trop. De São Paulo.

[8] Frank T.D., Carter A., Jahagirdar D., Biehl M.H., Douwes-Schultz D., Larson S.L., Arora M., Dwyer-Lindgren L., Steuben K.M., Abbastabar H. (2019). Global, regional, and national incidence, prevalence, and mortality of HIV, 1980–2017, and forecasts to 2030, for 195 countries and territories: A systematic analysis for the Global Burden of Diseases, Injuries, and Risk Factors Study 2017. Lancet HIV.

[9] Agholi M., Shahabadi S.N., Motazedian M.H., Hatam G.R. (2016). Prevalence of enteric protozoan oocysts with special reference to Sarcocystis cruzi among fecal samples of diarrheic immunodeficient patients in Iran. Korean J. Parasitol..

[10] Ahmadpour E., Safarpour H., Xiao L., Zarean M., Hatam-Nahavandi K., Barac A., Picot S., Rahimi M.T., Rubino S., Mahami-Oskouei M. (2020). Cryptosporidiosis in HIV-positive patients and related risk factors: A systematic review and meta-analysis. Parasite.

[11] Safarpour H., Cevik M., Zarean M., Barac A., Hatam-Nahavandi K., Rahimi M.T., Baghi H.B., Koshki T.J., Pagheh A.S., Shahrivar F. (2020). Global status of Toxoplasma gondii infection and associated risk factors in people living with HIV. Aids.

[12] Ahmadpour E., Ghanizadegan M.A., Razavi A., Kangari M., Seyfi R., Shahdust M., Yazdanian A., Safarpour H., Bannazadeh Baghi H., Zarean M. (2019). Strongyloides stercoralis infection in human immunodeficiency virus-infected patients and related risk factors: A systematic review and meta-analysis. Transbound. Emerg. Dis..

[13] Mathers C.D., Loncar D. (2006). Projections of global mortality and burden of disease from 2002 to 2030. PLoS Med..

[14] Akalu T.Y., Aynalem Y.A., Shiferaw W.S., Merkeb Alamneh Y., Getnet A., Abebaw A., Atnaf A., Abate A., Tilahun M., Kassie B. (2022). National burden of intestinal parasitic infections and its determinants among people living with HIV/AIDS on anti-retroviral therapy in Ethiopia: A systematic review and meta-analysis. SAGE Open Med..

[15] Assefa S., Erko B., Medhin G., Assefa Z., Shimelis T. (2009). Intestinal parasitic infections in relation to HIV/AIDS status, diarrhea and CD4 T-cell count. BMC Infect. Dis..

[16] Olopade B.O., Idowu C.O. (2017). Intestinal parasites among HIV-infected patients at obafemi awolowo university teaching hospitals complex, Ile-Ife. Ann. Trop. Pathol..

[17] Paboriboune P., Phoumindr N., Borel E., Sourinphoumy K., Phaxayaseng S., Luangkhot E., Sengphilom B., Vansilalom Y., Odermatt P., Delaporte E. (2014). Intestinal parasitic infections in HIV-infected patients, Lao People’s Democratic Republic. PLoS ONE.

[18] Requena I., Añez H., Lacourt E., Blanco Y., Castillo H., Rivera M., Devera R. (2007). Elevada prevalencia de coccidios intestinales en pacientes infectados con el Virus de la Inmunodeficiencia Humana en Ciudad Bolívar, Venezuela. Rev. Bioméd..

[19] Almeria S., Cinar H.N., Dubey J.P. (2019). *Cyclospora cayetanensis* and cyclosporiasis: An update. Microorganisms.

[20] Hadjilouka A., Tsaltas D. (2020). *Cyclospora cayetanensis*—Major outbreaks from ready to eat fresh fruits and vegetables. Foods.

[21] Chacín-Bonilla L. (2010). Epidemiology of *Cyclospora cayetanensis*: A review focusing in endemic areas. Acta Trop..

[22] Masoumi-Asl H., Khanaliha K., Bokharaei-Salim F., Esteghamati A., Kalantari S., Hosseinyrad M. (2019). Enteric opportunistic infection and the impact of antiretroviral therapy among hiv/aids patients from Tehran, Iran. Iran. J. Public Health.

[23] Ramezanzadeh S., Beloukas A., Pagheh A.S., Rahimi M.T., Hosseini S.A., Oliveira S.M.R., de Lourdes Pereira M., Ahmadpour E. (2022). Global Burden of *Cyclospora cayetanensis* Infection and Associated Risk Factors in People Living with HIV and/or AIDS. Viruses.

[24] Ortega Y.R., Sanchez R. (2010). Update on *Cyclospora cayetanensis*, a food-borne and waterborne parasite. Clin. Microbiol. Rev..

[25] Connor B.A. (2020). Cyclosporiasis. Hunter’s Tropical Medicine and Emerging Infectious Diseases.

[26] Wang Z.-D., Liu Q., Liu H.-H., Li S., Zhang L., Zhao Y.-K., Zhu X.-Q. (2018). Prevalence of Cryptosporidium, microsporidia and Isospora infection in HIV-infected people: A global systematic review and meta-analysis. Parasites Vectors.

[27] Dubey J., Almeria S. (2019). *Cystoisospora belli* infections in humans: The past 100 years. Parasitology.

[28] Tatfeng Y., Usuanlele M., Orukpe A., Digban A., Okodua M., Oviasogie F., Turay A. (2005). Mechanical transmission of pathogenic organisms: The role of cockroaches. J. Vector Borne Dis..

[29] Laksemi D., Suwanti L.T., Mufasirin M., Suastika K., Sudarmaja M. (2020). Opportunistic parasitic infections in patients with human immunodeficiency virus/acquired immunodeficiency syndrome: A review. Vet. World.

[30] Gebrecherkos T., Kebede H., Gelagay A.A. (2019). Intestinal parasites among HIV/AIDS patients attending University of Gondar Hospital, northwest Ethiopia. Ethiop. J. Health Dev..

[31] Agholi M., Hatam G.R., Motazedian M.H. (2013). HIV/AIDS-associated opportunistic protozoal diarrhea. AIDS Res. Hum. Retrovir..

[32] Taghipour H., Mosaferi M. (2009). Characterization of medical waste from hospitals in Tabriz, Iran. Sci. Total Environ..

[33] Garcia L.S., Procop G.W. (2016). Diagnostic medical parasitology. Manual of Commercial Methods in Clinical Microbiology: International Edition.

[34] Oguoma V., Ekwunife C. (2007). The need for a better method: Comparison of direct smear and formol-ether concentration techniques in diagnosing intestinal parasites. Internet J. Trop. Med..

[35] Lalonde L.F., Gajadhar A.A. (2008). Highly sensitive and specific PCR assay for reliable detection of *Cyclospora cayetanensis* oocysts. Appl. Environ. Microbiol..

[36] Tamura K., Dudley J., Nei M., Kumar S. (2007). MEGA4: Molecular evolutionary genetics analysis (MEGA) software version 4.0. Mol. Biol. Evol..

[37] Eisenberg J.N., Scott J.C., Porco T. (2007). Integrating disease control strategies: Balancing water sanitation and hygiene interventions to reduce diarrheal disease burden. Am. J. Public Health.

[38] Brown J., Cairncross S., Ensink J.H. (2013). Water, sanitation, hygiene and enteric infections in children. Arch. Dis. Child..

[39] World Health Organization (2010). WHO Recommendations on the Management of Diarrhoea and Pneumonia in HIV-Infected Infants and Children: Integrated Management of Childhood Illness (IMCI).

[40] Utami W.S., Murhandarwati E.H., Artama W.T., Kusnanto H. (2020). Cryptosporidium infection increases the risk for chronic diarrhea among people living with HIV in Southeast Asia: A systematic review and meta-analysis. Asia Pac. J. Public Health.

[41] Gendrel D., Treluyer J., Richard-Lenoble D. (2003). Parasitic diarrhea in normal and malnourished children. Fundam. Clin. Pharmacol..

[42] DuPont H.L. (2016). Persistent diarrhea: A clinical review. JAMA.

[43] Mahmoudvand H., Sepahvand A., Khatami M., Moayyedkazemi A. (2019). Prevalence and associated risk factors of *Cystoisospora belli* and *Cyclospora cayetanensis* infection among Iranian patients with colorectal cancer. J. Parasit. Dis..

[44] Opoku Y.K., Boampong J.N., Ayi I., Kwakye-Nuako G., Obiri-Yeboah D., Koranteng H., Ghartey-Kwansah G., Asare K.K. (2018). Socio-Behavioral Risk Factors Associated with Cryptosporidiosis in HIV/AIDS Patients Visiting the HIV Referral Clinic at Cape Coast Teaching Hospital, Ghana. Open AIDS J..

[45] Katiyar M., Gulati R., Pagal S., Rajkumari N., Singh R. (2021). Molecular detection of *Cystoisospora belli* by single-run polymerase chain reaction in stool samples. Indian J. Gastroenterol..

